# Production of Zebrafish Offspring from Cultured Female Germline Stem Cells

**DOI:** 10.1371/journal.pone.0062660

**Published:** 2013-05-03

**Authors:** Ten-Tsao Wong, Abraham Tesfamichael, Paul Collodi

**Affiliations:** Department of Animal Sciences, Purdue University, West Lafayette, Indiana, United States of America; National University of Singapore, Singapore

## Abstract

Zebrafish female germline stem cell (FGSC) cultures were generated from a transgenic line of fish that expresses Neo and DsRed under the control of the germ cell specific promoter, *ziwi* [*Tg(ziwi:neo);Tg(ziwi:DsRed)*]. Homogeneous FGSC cultures were established by G418 selection and continued to express *ziwi* for more than 6 weeks along with the germ cell markers *nanos3*, *dnd*, *dazl* and *vasa*. A key component of the cell culture system was the use of a feeder cell line that was initiated from ovaries of a transgenic line of fish [*Tg(gsdf:neo)*] that expresses Neo controlled by the zebrafish gonadal soma derived factor (*gsdf*) promoter. The feeder cell line was selected in G418 and engineered to express zebrafish leukemia inhibitory factor (Lif), basic fibroblast growth factor (Fgf2) and glial-cell-line derived neurotrophic factor (Gdnf). These factors were shown to significantly enhance FGSC growth, survival and germline competency in culture. Results from cell transplantation experiments revealed that the cultured FGSCs were able to successfully colonize the gonad of sterile recipient fish and generate functional gametes. Up to 20% of surviving recipient fish that were injected with the cultured FGSCs were fertile and generated multiple batches of normal offspring for at least 6 months. The FGSC cultures will provide an in vitro system for studies of zebrafish germ cell growth and differentiation and their high frequency of germline transmission following transplantation could form the basis of a stem cell-mediated strategy for gene transfer and manipulation of the zebrafish genome.

## Introduction

Fish are able to produce large numbers of eggs throughout their reproductive life and maintain a high level of fecundity [1]. Genetic studies of zebrafish [2] and clonal analysis of medaka [3] have provided evidence for the existence of a population of female germline stem cells (FGSCs) that is maintained in the adult fish ovary and is responsible for the continuous production of oocytes. The existence of fish FGSCs in the adult ovary has been further confirmed by cell transplantation studies with trout [4] and zebrafish [5] showing that donor ovarian germ cells can colonize the gonads of sterile recipient fish and produce functional gametes in the adult chimeras. Normal offspring derived from the transplanted cells were produced in each study. The latter studies were performed by directly transferring freshly isolated ovarian germ cells into recipients without first maintaining the cells in culture. The ability to propagate and manipulate the FGSCs in culture before transplanting them into a recipient would provide a cell-based strategy for gene transfer and targeting in the fish. Although the zebrafish is an exceptional model for studies in many research areas [6], [7], [8], [9], [10], [11], a stem cell-based approach to gene targeting is currently not available.

In this study, we have developed an approach using drug selection to isolate and culture zebrafish FGSCs that are obtained from a transgenic line of fish that expresses Neo under the control of a germ cell specific promoter. Using this strategy, we have established homogeneous cultures of zebrafish FGSCs that are able to proliferate for several weeks in culture and, after transplantation, successfully colonize the gonad of infertile recipient larvae to generate functional gametes. Zebrafish germline chimeras were generated at a high frequency and normal offspring derived from the transplanted FGSCs were produced.

## Materials and Methods

### Animals and Ethics

Zebrafish were maintained and staged as previously described [12]. All of the experimental procedures and protocols described in this study were approved by the Purdue University Animal Care and Use Committee and adhered to the National Research Council’s Guide for Care and Use of Laboratory Animals.

### Plasmid Construction

The gonadal soma derived factor (*gsdf*) genomic sequence was identified in Zebrafish chromosome 21 genomic scaffold, Zv9_scaffold2881 (NW_003335885). The following primers were used to amplify the 3.8-kb *gsdf* promoter (64926 to 61158 in NW_003335885) using forward Fwd1: 5′-GATCTCGAGGAGTGTTGCGCTGGTAATGTGT-3′ and reverse primer Rev1: 5′-ATCGGGCCCAGCATGTCTGTGGATTCAGGAGCG-3′. Zebrafish testicular genomic DNA was used for PCR amplification and the PCR program was: 94°C (1 minute), 30 cycles of 94°C (10 seconds)/68°C (5 minutes) and 68°C (10 minutes) using Advantage® Genomic LA Polymerase Mix (Clontech, Mountain View, CA, USA). The cloned and verified *gsdf* promoter fragment was assembled 5′ of *neo* to generate *gsdf-neo* expression construct. The *ziwi* promoter fragment obtained from Dr. Draper [13] was assembled 5′ of *neo* or *DsRed* to generate *ziwi-neo* and *ziwi-DsRed* expression constructs. All the constructs were sub-cloned into a modified Tol2pA [14] vector resulting in the expression cassette being flanked with Tol2 transposon sites to enhance the insertion of transgenic constructs into the zebrafish genome [15]. The resulting plasmids were designated pGsdf-neo, pZiwi-neo and pZiwi-DsRed.

To construct plasmid that expresses zebrafish leukemia inhibitory factor (Lif), the zebrafish *lif* cDNA [16] was sub-cloned into a vector that carried the puromycin resistant gene (*pac*) to generate pLif-pac plasmid. To construct plasmid that expresses zebrafish basic fibroblast growth factor (Fgf2), the cDNA of *fgf2* [16] was sub-cloned into pZeoSV2 (Invitrogen, Carlsbad, CA, USA) expression vector that carried the zeocin resistant gene (*zeo*) to generate pZeoSV2-Fgf2 plasmid. To construct plasmid that expresses zebrafish glial-cell-line derived neurotrophic factor (Gdnf), the primers Fwd2: 5′-ATGAAGTTATGGGACATTCTAGCC-3′ and Rev2: 5′-TCAAACGCAAGCACACTTTTTAGC-3′ were used to amplify cDNA that encodes zebrafish Gdnf from zebrafish testicular cDNA using Advantage® 2 PCR Kit (Clontech). PCR program was 94°C (1 min), 35 cycles of 94°C (10 sec)/60°C (10 sec)/68°C (1 min) and 68°C (6 min). The resulting PCR product was first cloned into pGEM-T-easy vector (Promega, Madison, WI, USA) and then sub-cloned into pZeoSV2 (Invitrogen) to generate pZeoSV2-Gdnf plasmid.

### Production of Transgenic Fish

To produce *Tg(gsdf:neo)*, *Tg(ziwi:neo)* and *Tg(ziwi:DsRed)* transgenic fish, 1 to 2 nl of a solution containing 7.5 ng/µl Tol2 RNA and 25 ng/µl of pGsdf-neo, pZiwi-neo or pZiwi-DsRed was injected into one- to two-cell stage embryos. The embryos were raised to adults and the male founders were identified by screening sperm samples for germline transmission of the transgenic construct. Sperm was collected from each male and analyzed by PCR using *gsdf* forward primer Fwd3: 5′-AGTGTAAAGTATTCCAAGGCCAAG-3′ and *neo* reverse primer Rev3: 5′-ATACTTTCTCGGCAGGAGCA-3′ for *Tg(gsdf:neo)*; *ziwi* forward primer Fwd4: 5′-CCCTTTACCAGTCCCAAGTCTGTT-3′ and *neo* reverse primer Rev3 for *Tg(ziwi:neo)* and *ziwi* forward primer Fwd4 and DsRed reverse primer Rev4: 5′-AGCCCATGGTCTTCTTCTGCATCA-3′ for *Tg(ziwi:DsRed)*. In our initial experiments, we identified 6 transgenic founders for *Tg(gsdf:neo)*, 5 transgenic founders for *Tg(ziwi:neo)* and 8 transgenic founders for *Tg(ziwi:DsRed)*. Three male founders from each line were chosen for further analysis and out-crossing to wild-type females to produce the stable lines. Genomic PCR screening was used to identify F1 and F2 offspring that carried *gsdf-neo*, *ziwi-neo* or *ziwi-DsRed* using the primers listed above. Tissue from fin-clip or 5 dpf individual embryo was used for genomic DNA extraction using a published protocol [2]. Double transgenic *Tg(ziwi:neo);Tg(ziwi:DsRed)* was produced by crossing *Tg(ziwi:neo)* with *Tg(ziwi:DsRed)*.

### Immunocytochemistry and Histology

Zebrafish were euthanized in 0.016% tricaine (ethyl-3-aminobenzoate methanesulfonic acid) (Sigma-Aldrich, St Louis, MO) solution in water, and gonads were removed and fixed with 4% paraformaldehyde in phosphate-buffered saline (PBS) at 4°C. For frozen section, after 2 hour fixation the tissue was rinsed two times in PBS and then immersed in sucrose solution (30% sucrose in PBS). The following day the samples were frozen with optimal cutting temperature compound (Sakura Finetek, Torrance, CA, USA) on dry ice, and serial cryostat sections (10 µm) were prepared using a Leica CM1850 cryostat (Leica, Buffalo Grove, IL, USA). The ovarian sections of *Tg(gsdf:neo)* were used to visualize the expression of Neo using a published protocol [17] with 1∶1000 dilution of mouse monoclonal IgG against neomycin phosphotransferase II (Abcam, Cambridge, MA) and a 1∶500 dilution of Alexa Fluor 488 AffiniPure goat anti-mouse IgG (Jackson ImmunoResearch Lab, Inc, West Groove, PA, USA). For whole mount immunocytochemical staining, after overnight fixation the tissue was washed two times PBS with 0.5% Triton X-100 (PBST), the fixed gonads were incubated in acetone at -20°C for 8 minutes. After another three 15-minute washes with PBST, the gonads were blocked for 1 hour at 25°C with antibody incubation buffer that contains 3% goat serum, 2% blocking reagent (Roche, Indianapolis, IN, USA) and 0.5% DMSO in PBST. Gonads were then incubated with a 1∶1000 dilution of mouse monoclonal IgG against neomycin phosphotransferase II (Abcam) and a 1∶3000 dilution of rabbit antiserum against zebrafish Vasa [18] at 4°C for overnight. After three 30-minute washes in PBST, gonads were then incubated with a 1∶500 dilution of Cy3 AffiniPure goat anti-rabbit IgG and a 1∶500 dilution of Alexa Fluor 488 AffiniPure goat anti-mouse IgG (Jackson ImmunoResearch Lab, Inc.) in antibody incubation buffer at 4°C for overnight. Excess antibody was removed by 1 hour wash with PBST and 300 nM DAPI and three 30-minute washes with PBST. The expression of Neo or Vasa in the gonads of *Tg(gsdf:neo)* and *Tg(ziwi:neo)* were visualized using a Nikon Eclipse TE200 fluorescence microscope (Nikon, Tokyo, Japan) equipped with a RT Slider digital camera (Spot Imaging Solution, Sterling Heights, MI, USA). For histology, the sections were stained with hematoxylin-eosin and examined by light microscopy.

### Production of Feeder Cells

Ovaries combined from four to five 3-month-old *Tg(gsdf:neo)* zebrafish were minced with scissors and dissociated with 0.2% collagenase (Invitrogen) in PBS at 28.5°C for 1 hour. The resulting ovarian cell suspension was filtered through a 60-µm mesh to remove large debris and oocytes, washed twice with Leibowitz’s L-15 medium (Sigma-Aldrich) and re-suspended in 3 ml of L-15. The cell suspension was transferred into 100 mm tissue culture dishes in L-15 medium with 3 mg/ml of D-(+)-glucose (Sigma-Aldrich). After the cells attached, the FBS (Harlan, Indianapolis, IN, USA) were added into dishes at the final concentration of 10%. The cells were cultured at 28.5°C with G418 (300 µg/ml, Invitrogen) and the medium was replaced every 4 to 5 days. Individual colony was harvested, expanded and continuously selected with G418 to generate immortalized ovarian cell lines as candidate feeder cells to be tested with the FGSC cultures. To generate the feeder cell lines that express zebrafish growth factors, one of immortalized ovarian cell line was further transfected with growth factor expression plasmids pLif-pac, pZeoSV2-Fgf2 or pZeoSV2-Gdnf using GenePulser Xcell electroporator (Bio-Rad, Hercules, CA, USA) at 950 µF/275 V in a 0.4 cm cuvette. The cells were selected with either 2 µg/ml puromycin (Sigma-Aldrich) and/or 100 µg/ml zeocin (Invitrogen) to establish growth factor expressing feeder lines. Conditioned medium from each cell line was collected from confluent cultures every 3 days and stored at –20°C before use.

### FGSC Culture and Growth Assay

To evaluate the growth-promoting activity of each candidate feeder line on the FGSC cultures, 2×10^5^ cells of each feeder line were seeded into individual well of a 12-well plate in triplicate. The candidate feeder cells were irradiated for 8 Krad to generate growth-arrested feeder cells. The FGSCs isolated from ovaries of 18 *Tg(ziwi:neo);Tg(ziwi:DsRed)* transgenic fish (10–12 weeks old) using a published protocol [5] were seeded into 12 wells (in average, 1.5 fish/well) and cultured with a modified StemPro®-34 SFM culture medium (Invitrogen) with the addition of minimal essential medium (MEM) vitamin solution (Invitrogen), MEM nonessential amino acid solution (Invitrogen), 5% KnockOut™ serum replacement (Invitrogen), 0.4% BSA (Sigma-Aldrich), 10 µg/ml insulin (Sigma-Aldrich), 100 µg/ml transferrin (Sigma-Aldrich), 60 µM putrescine (Sigma-Aldrich), 30 nM sodium selenite (Sigma-Aldrich), 3 mg/ml D-(+)-glucose (Sigma-Aldrich), 1 µl/ml DL-lactic acid (Sigma-Aldrich), 2 mM l-glutamine (Sigma-Aldrich), 50 µM 2-mercaptoethanol (Sigma-Aldrich), 100 µM ascorbic acid (Sigma-Aldrich), 10 µg/ml d-biotin (Sigma-Aldrich), 30 ng/ml β-estradiol (Sigma-Aldrich), 60 ng/ml progesterone (Sigma-Aldrich), 2 µM retinol (Sigma-Aldrich), 120 µg/ml penicillin G, 25 µg/ml ampicillin and 200 µg/ml streptomycin sulfate (Sigma-Aldrich), 40 ng/ml human epidermal growth factor (EGF) (StemGent, Cambridge, MA, USA), 10 ng/ml human FGF2 (StemGent), 1000 U/ml murine LIF (StemGent), 10 ng/ml human GDNF (PeproTech, Rocky Hill, NJ, USA), 20 ng/ml human fibroblast growth factor 9 (FGF9) (PeproTech); 1% FBS (Harlan), 1% fish serum (East Coast Biologics, Inc., North Berwick, ME, USA) and 30% conditioned medium. When using zebrafish growth factor (Lif, Fgf2, Gdnf) expressing cells as feeder cells, the correspondence mammalian recombinant growth factor was omitted from medium. The cells were maintained at 28.5°C in an atmosphere of 3% carbon dioxide and the medium was replaced every 3–4 days. After three weeks of culture, G418 selected and DsRed expressing colonies and cells in each individual well were counted using a Nikon Eclipse TE200 fluorescence microscope. For continuing culturing, the 1/3 of fresh growth-arrested feeder cells was added into culture after the first 3 weeks of culture or the FGSCs were harvested and passed to a new plate that contained fresh growth-arrested feeder cells. The FGSC colonies do not attached to feeder cells tightly and over 85% of them can be easily disassociated with 0.25% EDTA in PBS. After EDTA disassociation, the FGSCs were transferred to a tube and washed with PBS and culture medium. Additional 1 to 2 minutes trypsin (0.25%, Invitrogen) incubation was used to recover the rest of FGSCs after the EDTA disassociation.

### RNA Extraction and RT-PCR Analysis

Total RNA was prepared from cultured FGSCs using Trizol reagent (Invitrogen) followed by DNase treatment (Ambiom, Austin, TX, USA). The cDNA was synthesized using MMLV-RT (Promega) according to manufacturer’s instructions. The sequences of the primers used for PCR analysis were: *dazl* (Fwd5: 5′- TACCCGTGTGCCTGATATGTGGTT-3′/Rev5: 5′- AGGGTTAGCAAAGTCTGCACTCCA-3′); *dnd* (Fwd6: 5′- TCTGCAGGAATGGATGCAGAGGAA-3′/Rev6: 5′- TCTGACGGTGATGGAAATGCCGTA-3′); *nanos3* (Fwd7: 5′- AGCCTTGGAAGGACTACATGGGTT-3′/Rev7: 5′- TGATTTGGCGTACACCGAGCAGTA-3′); *vasa* (Fwd8: 5′-GCAGGACCCAAGGTTGTTTA-3′/Rev8: 5′-GCACTTTACTCAGGCCAATCT-3′); *ziwi* (Fwd9: 5′- CTCAGAGGTTTAGAACTACGTGAGGG-3′/Rev9: 5′- GTGGGATGTTGAATGGGTCATCAGGA-3′); *βactin* (Fwd10: 5′-AGACATCAGGGTGTCATGGTTGGT-3′/Rev10: 5′-TGGTCTCGTGGATACCGCAAGATT-3′). The PCR program was 94°C (1 minute), 35 cycles at 94°C (10 seconds)/55°C (10 seconds)/68°C (1 minute).

### Ovarian Germ Cell Transplantation and Analysis

To prepare infertile recipient larvae, wild-type embryos at the 1-cell-stage were injected with *dnd* antisense morpholino (MO) at 1 µg/µl in 0.06% phenol red solution to deplete the endogenous germ cell population. The *dnd* MO solution was injected directly into the cytoplasm of the embryos. In order to ensure that all the recipients used for transplantation were infertile, only the embryos that contained phenol red in all of the cells at the 4- to 8-cell-stage were selected and raised to be used as recipients. Cultured FGSCs were collected with 0.25% EDTA in PBS, washed, re-suspended in 50 µl of L-15 medium and immediately transplanted into two-week-old zebrafish larvae that had been treated with a *dnd* antisense MO [19]. Cell transplantations were performed under a stereomicroscope using a glass micropipette needle. Cultured FGSCs were transplanted into the abdominal cavity under the swim bladder and close to the gonads of the recipient according to a published method [5]. Two weeks after transplantation, the recipients were examined by fluorescence microscopy and the potential germline chimeras were identified based on the presence of DsRed-positive cells in the gonadal region. To determine if the transplanted cells were able to generate functional gametes, the recipient fish were raised to sexual maturity and paired with wild-type zebrafish mates.

### Statistical Analysis

Data obtained from growth assay were presented as the mean and standard deviation. For statistical analysis Student t tests or one-way ANOVA were applied followed by Bonferroni–Dunn tests using SAS program. The significance was accepted at p<0.05.

## Results

### Production of *Tg(ziwi:neo);Tg(ziwi:DsRed)* Double Transgenic Fish

**Figure 1:**
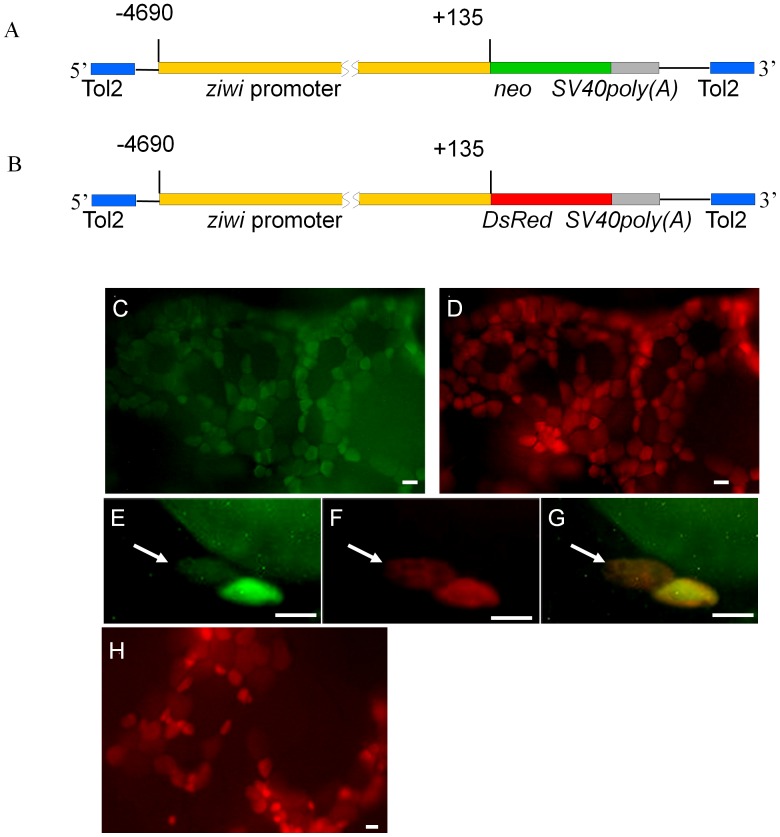
Production of *Tg(ziwi:neo)* and *Tg(ziwi:DsRed)* transgenic fish. (A,B) Diagram of the constructs used to produce the *Tg(ziwi:neo)* and *Tg(ziwi:DsRed)* transgenic fish. A 4.8 kb fragment of the *ziwi* promoter [13] was used to drive expression of (A) *neo* and (B) *DsRed*. (C) Neo (green) and (D) Vasa (red) are expressed in the same ovarian germ cells of *Tg(ziwi:neo)*. Higher magnification showing expression of (E) Neo (green) and (F) Vasa (red) in the oogonia (white arrows) of *Tg(ziwi:neo)*; (G) merged photo of E and F. (H) DsRed was detected in the ovarian germ cells of *Tg(ziwi:DsRed)*. Scale bar = 50 µm for C, D, H and 20 µm for E, F, G.

To establish FGSC cultures, we produced *Tg(ziwi:neo);Tg(ziwi:DsRed)* double transgenic zebrafish in which the FGSCs express Neo and DsRed. A 4.8-kb fragment of the *ziwi* promoter, previously shown to direct EGFP expression in ovarian germ cells including oogonial stem cells [13], was used (Fig. 1A, B) to generate the *Tg(ziwi:neo)* and *Tg(ziwi:DsRed)* transgenic line of fish. Immunocytochemical analysis of ovarian tissue dissected from the transgenic fish confirmed that Neo is expressed in the Vasa-positive ovarian germ cells including oogonia (Fig. 1C–G). Fluorescence microscopy revealed the DsRed is also expressed in ovarian germ cells (Fig. 1H) of *Tg(ziwi:DsRed)*.

### Derivation of FGSC Cultures Initiated from *Tg(ziwi:neo);Tg(ziwi:DsRed)* Fish by G418 Selection

**Figure 2:**
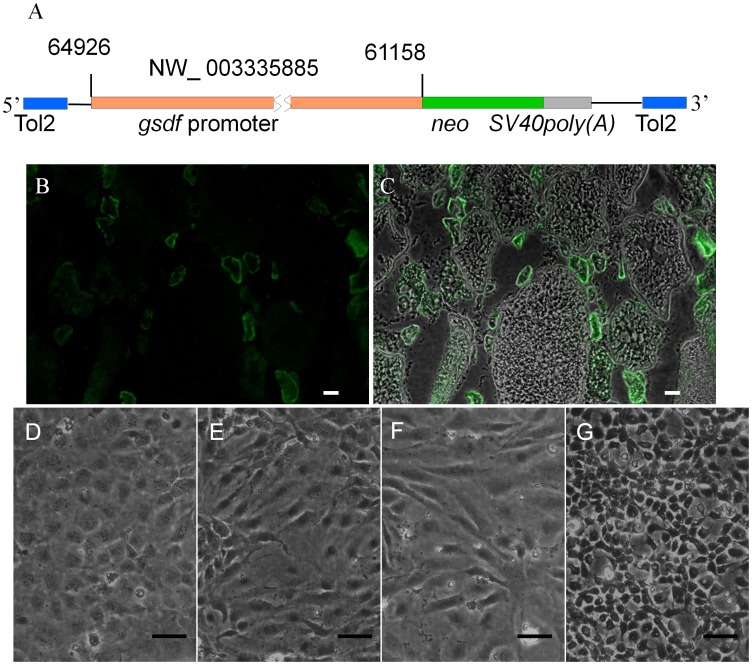
Production of ovarian-somatic feeder cell (OFC) lines. (A) Diagram of the construct used to produce the *Tg(gsdf:neo)* fish. The plasmid contained a 3.8 kb *gsdf* promoter controlling expression of *neo*. (B) Neo (green) expressed in ovarian somatic cells, particularly the granulosa cells, of *Tg(gsdf:neo)*. (C) The merger of (B) with the corresponding bright field photo. Photomicrograph showing 12 month-old (D) OFC1, (E) OFC3, (F) OFC5 and (G) OFC6 ovarian feeder cell cultures. Scale bar = 50 µm.

**Figure 3:**
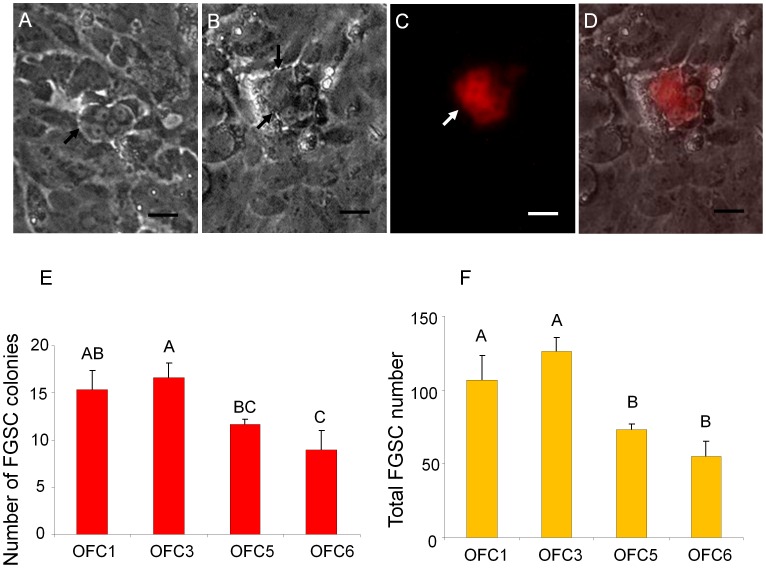
Effect of OFC feeder lines on FGSC cultures. Photomicrograph showing G418-selected FGSCs that were initiated from *Tg(ziwi:neo);Tg(ziwi:DsRed)* zebrafish and grown on OFC feeders showing a (A) four-cell and (B) eight-cell colonies, black arrows; (C) DsRed expression in the eight-cell colony; (D) merged images of B and C. (E) OFC3 significantly (p = 0.002) promoted FGSC colony formation. (F) Both OFC1 and OFC3 significantly (p = 0.0002) increased the number of FGSCs in a 3-week culture. Data points not sharing a letter (A, B, C) are significantly different by Bonferroni–Dunn tests. Scale bar = 20 µm.

Ovarian tissue dissected from 10- to 12-week old *Tg(ziwi:neo);Tg(ziwi:DsRed)* zebrafish was dissociated and partially purified through a Percoll gradient to obtain a cell fraction enriched for DsRed-positive ovarian germ cells [5]. Primary cell cultures were initiated from the cell fraction and *ziwi-neo*-expressing FGSCs were selected in G418. To propagate the FGSCs in culture, the cells were maintained on a growth-arrested feeder layer comprised of an ovarian-somatic feeder cell (OFC) line that was derived from *Tg(gsdf:neo)* transgenic zebrafish. The *Tg(gsdf:neo)* fish were generated using a 3.8-kb fragment of the *gsdf* promoter to drive *neo* expression (Fig. 2A) specifically in ovarian somatic cells of the transgenic fish (Fig. 2B, C). The *gsdf-neo*-expressing cell lines were obtained by G418 selection of ovarian primary cultures that were initiated from the *Tg(gsdf:neo)* fish. Four G418-resistant colonies (OFC1,3,5,6) were expanded to individual cell lines (Fig. 2D–G) and evaluated for use as feeder layers in FGSC cultures. DsRed-positive FGSCs, obtained by Percoll fractionation of *Tg(ziwi:neo);Tg(ziwi:DsRed)* ovarian tissue, were plated at clonal density onto a confluent layer of each feeder cell line and the growth of the FGSC clones were evaluated after 3 weeks. The results showed that a feeder layer consisting of either OFC1 or OFC3 promoted the proliferation of the FGSCs resulting in colonies of 4 or more G418-resistant and DsRed-positive cells (Fig. 3A–D). The morphology of the FGSCs in culture resembled the mitotically active oogonia found in the ovary [5] possessing a diameter of approximately 10 µm or less and a high nucleus-to-cytoplasm ratio with one to two nucleoli (Fig. 3A, B). Even after more than 3 weeks in culture on the ovarian cell feeder layer the FGSC colonies continued to express ziwi-DsRed (Fig. 3C). Although a few larger DsRed-positive ovarian germ cells resembling early-stage oocytes were initially observed in the cultures, these larger cells did not attach tightly to the feeder layer or proliferate in culture and were not present after 3 weeks. Most of the DsRed-positive cells were located within colonies and possessed an oogonium-like morphology. Since the results showed that OFC3 promoted FGSC colony formation and growth (Fig. 3E,F), this cell line was used for subsequent experiments.

**Figure 4:**
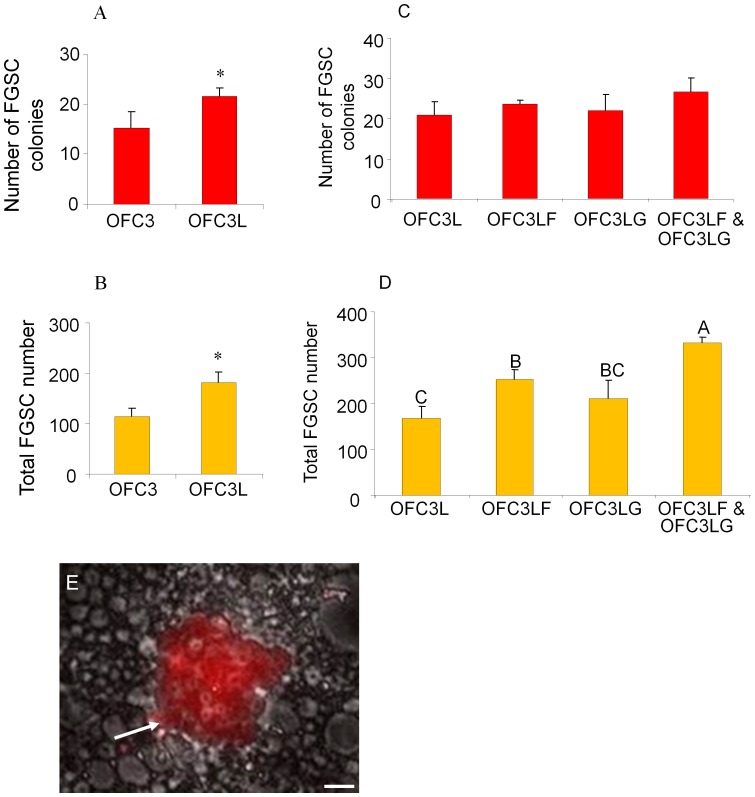
Effect of feeder cells expressing zebrafish Lif, Fgf2 and Gdnf on 3-week FGSC cultures. The presence of feeder cells expressing zebrafish Lif (OFC3L) significantly increased (A) FGSC colony number (p = 0.037) and (B) total FGSC number (p = 0.002) in 3-week cultures (* indicates a significant difference based on Student t test). (C) The presence of feeder cells expressing zebrafish Lif and Fgf2 (OFC3LF) or Lif and Gdnf (OFC3LG) did not significantly increase FGSC colony formation. (D) Addition of feeder cells expressing all 3 zebrafish factors (OFC3LF and OFC3LG) did significantly (p = 0.004) promote FGSC proliferation. (E) Photomicrograph showing a DsRed-positive (white arrow) FGSC colony containing about 28 cells that is growing on OFC3LF and OFC3LG. Data points not sharing a letter (A, B, C) are significantly different by Bonferroni–Dunn tests. Scale bar = 20 µm.

**Figure 5:**
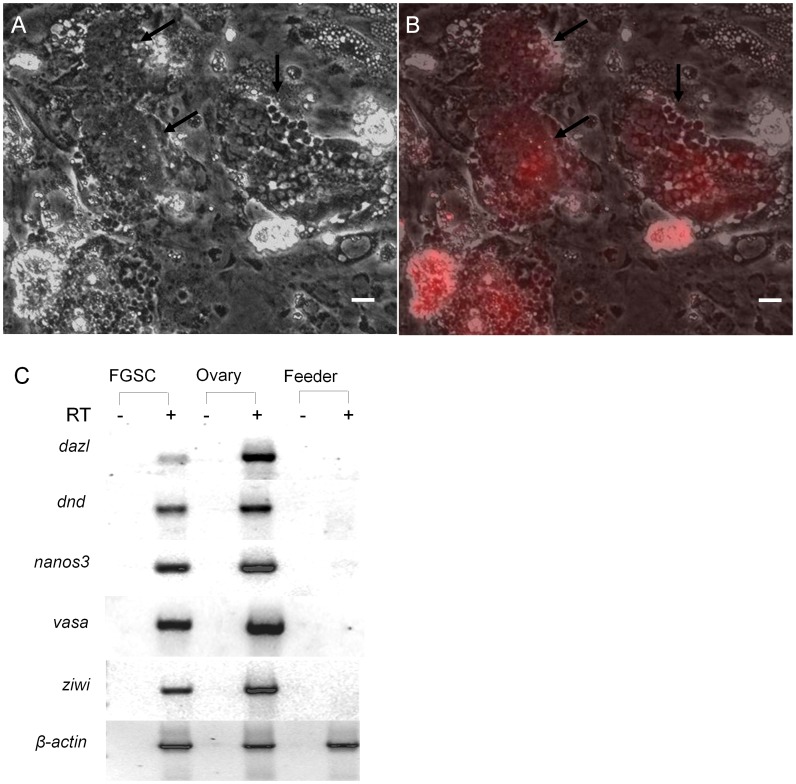
Effect of feeder cells expressing zebrafish Lif, Fgf2 and Gdnf on FGSCs in 6-week cultures. Photomicrographs showing two DsRed-positive FGSC colonies containing 100 or more cells (black arrows) under (A) bright field and (B) merged with UV (DsRed). (C) RT-PCR analysis of RNA isolated from 6-week FGSC cultures showing expression of germ cell specific maker genes, *dazl*, *dnd*, *nanos3*, *vasa* and *ziwi*. RT: reverse transcription; Scale bar = 20 µm.

To optimize the FGSC culture conditions, OFC3 was transfected with a plasmid encoding zebrafish Lif and stable colonies (OFC3L) that expressed the factor were isolated. Results showed that both FGSC colony formation and cell proliferation were significantly enhanced in the presence of OFC3L compared to cultures grown on the parent OFC3 feeder line and supplemented with recombinant mammalian LIF (Fig. 4A, B). To further optimize the FGSC culture conditions, the OFC3L feeder cells were engineered to stably express either zebrafish Gdnf (OFC3LG) or Fgf2 (OFC3LF) along with Lif and the mitogenic activity of each feeder line was tested. Although there was no significant effect of OFC3LG or OFC3LF feeder layers on the number of FGSC colonies that formed in culture (Fig. 4C), the presence of OFC3LF did enhance FGSC proliferation resulting in larger colonies (Fig. 4D) after 3 weeks of culture. The use of a feeder layer comprised of both OFC3LF and OFC3LG (1∶1 mix) resulted in optimal FGSC proliferation after 3 weeks in culture (Fig. 4D, E). After 6 weeks, the total number of FGSC colonies significantly decreased on each of the feeder layers when compared to the 3 week cultures (Table S1 and Fig. S1A); however, the remaining colonies were larger and total number of cells contained in each well increased. In the wells containing OFC3LF and OFC3LG feeder layers, the total number of FGSCs increased approximately 3-fold during this period (Fig. S1B). The largest colonies examined in the 6-week-old cultures contained up to 100 FGSCs (Fig. 5A,B) and the average number of FGSCs per colony increased 4-fold compared to the 3-week-old cultures (Fig. S1C). The results indicated that the average doubling time of the FGSCs on OFC3LF and OFC3LG feeder layers was approximately 10 days. RT-PCR analysis revealed that at 6-weeks the cultures continued to express germ cell-specific markers, *dazl*, *dnd*, *nanos3*, *vasa* and *ziwi* (Fig. 5C). In cultures maintained on OFC3 feeder cells and supplemented with recombinant mammalian LIF, FGF2 or GDNF, the number of FGSCs significantly decreased over 3 weeks and were nearly absent by 6 weeks (Fig. S1B) demonstrating that the recombinant mammalian growth factors were not able to sustain the FGSCs in culture.

### Cultured FGSCs were Able to Colonize the Gonads of Recipient Fish and Produce Functional Gametes following Transplantation

**Figure 6:**
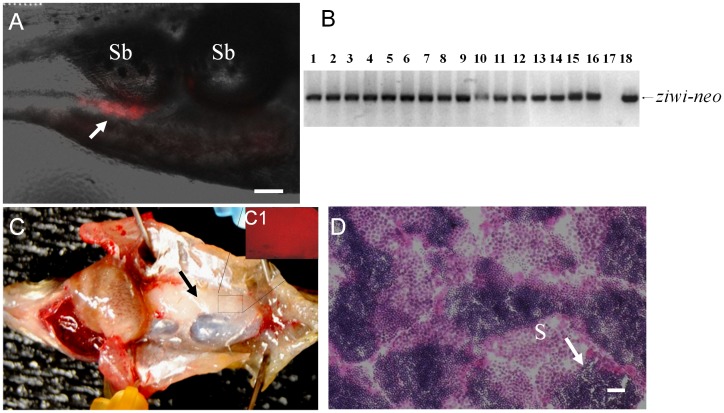
Germline transmission of 6-week cultured FGSCs. (A) Photomicrograph showing the incorporation of DsRed-positive cultured FGSCs (arrow) into the gonad of a recipient larva two weeks after transplantation. (B) Results of genomic PCR showing the presence of *ziwi-neo* sequences that were inherited by all of the F1 individuals (lanes 1 to 16) produced by a germline chimeric father. Negative control: genomic DNA template from a wild-type larva (lane 17); positive control: pZiwi-neo plasmid DNA template (lane 18). (C) Dissection of a fertile adult male recipient fish showing that the transplanted DsRed-positive FGSCs have proliferated and directed the formation of a testis (arrow) on one side of the body. (C1) Inset shows the gonad under UV light revealing the presence of DsRed-positive cells. (D) Transverse section of testis from a fertile recipient fish showing active spermatogenesis. S: spermatozoa. Scale bar = 50 µm for A, C and 20 µm for D.

Cell transplantation experiments were performed to evaluate the germline competency of the cultured FGSCs. The FGSCs obtained from 3- or 6-week-old cultures were transplanted into 2-week-old recipient larvae that had been treated with a *dnd* antisense MO to block endogenous germ cell formation [19]. Approximately 20–40 FGSCs were transplanted into each larva and two weeks after transplantation, the recipients were examined by fluorescence microscopy for the presence of DsRed-positive cells in the gonadal region (Fig. 6A). All the recipients were raised to sexual maturity and paired with wild-type zebrafish mates. A total of fifty adult recipients were obtained from three independent transplantation experiments using 3-week-old FGSC cultures. All 50 of the adult fish were found to be males and were bred pairwise with wild-type zebrafish females. A total of 10 recipients (20%) reproduced and generated normal offspring (Table 1). For cell transplantations using the 6-week-old FGSC cultures, a total of 43 adult recipients (all males) were obtained from three independent experiments and 7 of the recipients (16%) reproduced and generated normal offspring (Table 2). Genomic PCR analysis showed that all of the offspring inherited the *ziwi-neo* construct donated by the transplanted FGSCs (Fig. 6B). The fertile recipient fish produced multiple batches of normal F1 embryos for a period of at least 6 months, indicating that the transplanted FGSCs successfully colonized the recipient gonad and continued to generate functional gametes resulting in the permanent rescue of fertility in the sterile host. Dissection of 3 fertile individuals that were transplanted with 6-week-old FGSC cultures revealed that each fish possessed a single fully formed testis that carried DsRed-positive cells (Fig. 6C and C1). Histological examination of the fully-developed testis from 10 month-old fertile recipient fish revealed the presence of large numbers of mature sperm along with spermatogonial cells at different developmental stages present on individual tissue sections (Fig. 6D).

**Table 1:** Results from three transplantation (T) experiments using 3-week-old cultured FGSCs introduced into two-week-old sterile recipients.

Exp. groups	Number of recipients transplanted	Number of recipients thatsurvived to adulthood	Number and (%) of recipients carrying germline transmitted ovarian germ cells
T1	17	13	3 (23%)
T2	28	19	4 (21%)
T3	23	18	3 (17%)
Total	68	50	10 (20%)

**Table 2:** Results from three transplantation (T) experiments using 6-week-old cultured FGSCs introduced into two-week-old sterile recipients.

Exp. groups	Number of recipients transplanted	Number of recipients thatsurvived to adulthood	Number and (%) of recipients carrying germline transmitted ovarian germ cells
T1	15	9	1 (11%)
T2	16	12	2 (17%)
T3	29	22	4 (18%)
Total	60	43	7 (16%)

An F1 generation was obtained from founder fish that were produced by transplanting 6-week-old FGSC cultures. A total of 57 F1 individuals (34 females and 23 males) were raised to sexual maturity and gonads were dissected from 6 males and 6 females. The results showed that all 12 of the F1 fish possessed a pair of fully developed gonads containing DsRed-positive germ cells (data not shown). The remaining F1 fish were bred to produce normal F2 offspring. A flow chart diagram of the FGSC transplantations, screenings and analyses is shown in Fig. S2.

## Discussion

In this study, we established FGSC cultures initiated from the ovaries of *Tg(ziwi:neo);Tg(ziwi:DsRed)* transgenic zebrafish. Ziwi is an ortholog of *Drosophila* Piwi, an Argonaut-class protein that has been shown to be required for the maintenance of germline stem cells in *Drosophila* and *C. elegans* [20], [21]. In zebrafish, loss of Ziwi function results in a progressive decrease in the number of germ cells due to apoptosis during early development [22]. Germ cell number was significantly reduced in 3-week-old *ziwi* mutant zebrafish larvae and completely absent in 40-day-old fish. Analysis of the mutant fish indicated that Ziwi functioned to prevent germ cell apoptosis in a dose dependent manner [22]. Since *ziwi* is specifically expressed in the germ cell lineage of male and female zebrafish [13], [22], [23], it provided an ideal promoter to direct Neo and DsRed expression to the FGSCs. We used G418 selection of primary cell cultures initiated from *Tg(ziwi:neo);Tg(ziwi:DsRed)* ovarian tissue to isolate homogeneous populations of mitotically active FGSCs. In the presence of G418 ovarian somatic cells were eliminated and G418-resistant non-proliferating germ cells that were initially present in the culture were lost during medium change or passage. After several weeks the cultures contained colonies of proliferating DsRed-positive cells that exhibited an oogonium-like morphology.

A drug selection approach was also used to obtain an ovarian feeder cell line that promoted the growth and survival of FGSCs in culture. The *gsdf* promoter was used to drive Neo expression during selection of the feeder cell line. Gsdf is a transforming growth factor-beta (TGF-β)-related peptide that is expressed specifically in somatic cells that surround early-stage germ cells in the fish gonad [24], [25], [26]. Functional studies have revealed that Gsdf plays an important role in germ cell development and proliferation. In trout, Gsdf promotes the growth of primordial germ cells (PGCs) in the developing embryo and enhances spermatogonia proliferation in culture [25]. The decision to use the *gsdf* promoter to select for a suitable feeder cell line for FGSC culture was based on the presumption that the gonadal cells that express Gsdf most likely also produce additional factors that promote germ cell growth and survival. The results demonstrate that the drug selected ovarian feeder cell line was a crucial component of the FGSC culture system.

To optimize the mitogenic effect of the ovarian feeder cells, OFC3 was transfected to express zebrafish Lif, Fgf2 and Gdnf. Each of these factors have been shown to promote in vitro growth of mammalian germline stem cells [27], [28], [29], [30], [31], [32], [33], [34], [35], [36], [37], [38], [39], [40]. As in other studies involving fish germline cell cultures, our attempts to supplement the FGSC culture medium with the recombinant mammalian growth factors were not successful [41]. The use of OFC3 feeder cells expressing zebrafish Lif was sufficient to significantly increase the number of FGSC colonies indicating that Lif is required to support colony initiation and low-density cell growth. This result is consistent with a previous study reporting that LIF enhances the formation of mouse spermatogonial stem cell colonies in culture [34]. Our results show that once the FGSC colonies were initiated, optimal cell proliferation within each colony was achieved when the feeder cells expressed zebrafish Fgf2 and Gdnf in addition to Lif. After 6 weeks, colonies of at least 100 cells were found only in the cultures supplemented with all three zebrafish factors indicating that their combination is required for extended FGSC proliferation. A significant decrease in FGSC colony number was observed in the 6-week-old cultures although the colonies that remained were larger and the total number of FGSCs increased 3-fold. The general decrease in colony number may be the result of spontaneous differentiation and associated loss of cell proliferation in some of the colonies.

The effect of zebrafish Lif, Fgf2 and Gdnf on promoting in vitro proliferation and survival of the zebrafish FGSCs is consistent with results that have been obtained with germ cell cultures from other species. LIF is an important component of mouse spermatogonial cell [34] and PGC [30] cultures. FGF2 plays an important role in self-renewal of mouse spermatogonial stem cells [39] and is critical for the reprogramming of mouse PGCs to the EG cell phenotype [42]. GDNF has been shown to promote the proliferation of mouse spermatogonial cells in vivo and in culture [35], [36], [37], [38], [39]. Over-expression of GDNF in mouse testes stimulated the growth of spermatogonial cell proliferation and blocked spermatocyte differentiation [35]. Recombinant GDNF has also been used as a supplement in mammalian ovarian germline stem cell cultures [33]. In our studies supplementation of the zebrafish FGSC cultures with recombinant mammalian LIF, FGF2 and GDNF did not produce a mitogenic effect on the cells when compared to zebrafish growth factors expressed in feeder cells. The observed difference in biological activity of the growth factors could be due to species specificity of the zebrafish factors acting on zebrafish FGSCs or it may be due to the factors being synthesized by the zebrafish feeder cells instead of bacteria.

Using the culture conditions that were established in this study, the zebrafish FGSCs continued to proliferate and maintain expression of the germ-cell markers *dazl*, *dnd*, *nanos3*, *vasa* and *ziwi* for at least 6 weeks in vitro. Continuous expression of the germ cell markers along with the capacity to direct the formation of functional gonads in a large percentage of recipient fish following transplantation are evidence that the FGSCs maintained germ cell characteristics and germline competency in culture. The use of infertile recipients provided an effective strategy to efficiently screen for successful germline transmission of the transplanted FGSCs. Successful colonization of the recipient gonad resulted in the production of a fertile fish from which 100% of the F1 individuals were derived from the DsRed-positive transplanted FGSCs. Unsuccessful germline transmission resulted in an infertile adult fish. This strategy makes it convenient to distinguish the rare instance where MO-treatment of the embryos may fail to deplete the endogenous population of germ cells resulting in the development of a fertile fish, since the F1 population would lack DsRed donated by the FGSCs. In this study we did not obtain any MO-treated recipients that remained fertile without incorporating donor cells.

All of the fertile recipients obtained in this study were able to produce multiple batches of normal F1 embryos for at least 6 months. The recurrent production of functional gametes by the recipient fish indicates that the transplanted FGSCs directed the formation of a normal testis and successfully established the entire spermatogonial cell lineage in the recipient gonad consistent with the transplanted cells possessing a stem cell phenotype.

In this study, successful germline transmission of the transplanted cells resulted in the production of only male fertile recipients even though cultured ovarian cells were being transplanted. A similar result was obtained in two previous studies that reported only fertile male zebrafish were obtained when recipient larvae were transplanted with a single PGC [43] or freshly isolated ovarian germ cells [5]. A recent study has shown that depletion of oocytes, without removal of oogonia, in the ovary of adult zebrafish induced sex reversal to a sperm-producing male [44]. This result indicates that signals from female germ cells, particularly the oocytes, may be needed to promote or maintain female development. In our study, the transplanted FGSCs may not have produced a sufficient amount of the factor that is required to promote female development resulting exclusively in the production of fertile male recipient fish.

## Supporting Information

Figure S1 **Effect of feeder cells expressing zebrafish Lif, Fgf2 and Gdnf on 3-week and 6-week FGSC cultures.** After 6 weeks of culture, (A) the total number of FGSC colonies significantly decreased on each of the feeder layers when compared to the 3 week cultures; however, the remaining colonies were larger. (B) In the wells containing OFC3LF and OFC3LG feeder layers, the total number of FGSCs increased approximately 3-fold during this period, and (C) the average number of FGSCs per colony increased 4-fold compared to the 3-week-old cultures. (* indicates the significant difference by Student t tests).
(TIF)

Figure S2 **A flow chart diagram of the FGSC transplantations, screenings and analyses.** All adult recipients (F0) were found to be males and were able to induce wild-type (Wt) females to spawn eggs; about 16% to 20% of recipients were able to produce fertilized eggs. F1 offspring were screened to confirm the presence of *ziwi-neo* using PCR (Fig. 6B). An F1 generation produced by transplanting 6-week-old FGSC cultures was raised to adult and confirmed that normal and healthy F1 male and female can be obtained. Normal F2 offspring were produced by crossing F1 males with F1 females.
(TIF)

Table S1 Effect of feeder cells expressing zebrafish Lif, Fgf2 and Gdnf on 3-week and 6-week FGSC cultures. Number of FGSCs in each individual colony among 5 different culture conditions was presented.
(DOC)
