# Supplementary figures and images for: Production of Zebrafish Offspring from Cultured Female Germline Stem Cells
Source: PLoS One. 2013 May 3;8(5):e62660. doi: 10.1371/journal.pone.0062660 (PMC3643964; doi:10.1371/journal.pone.0062660)

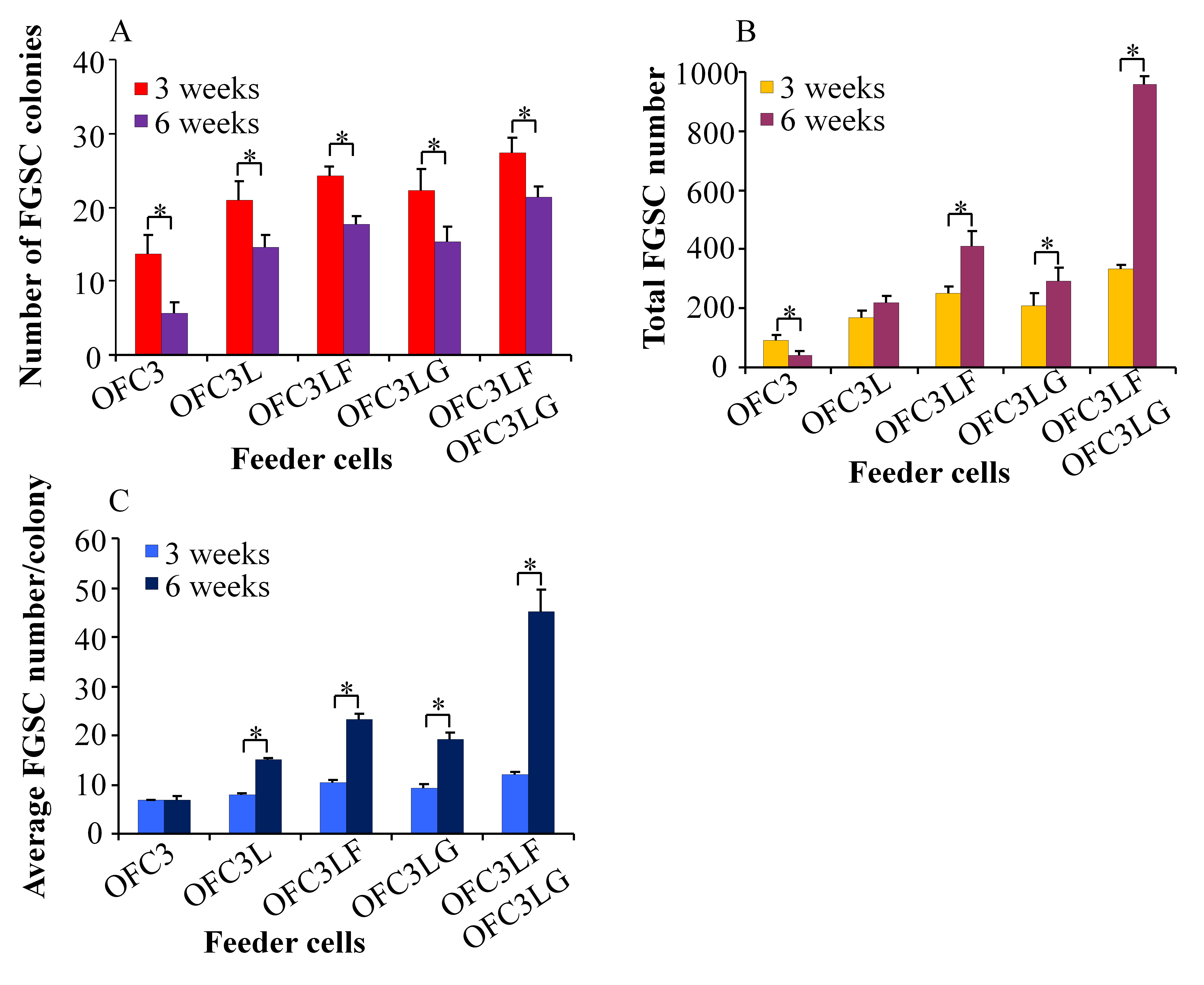

Supplement: Figure S1 — Effect of feeder cells expressing zebrafish Lif, Fgf2 and Gdnf on 3-week and 6-week FGSC cultures. After 6 weeks of culture, (A) the total number of FGSC colonies significantly decreased on each of the feeder layers when compared to the 3 week cultures; however, the remaining colonies were larger. (B) In the wells containing OFC3LF and OFC3LG feeder layers, the total number of FGSCs increased approximately 3-fold during this period, and (C) the average number of FGSCs per colony increased 4-fold compared to the 3-week-old cultures. (* indicates the significant difference by Student t tests). (TIF) [file pone.0062660.s001.tif]

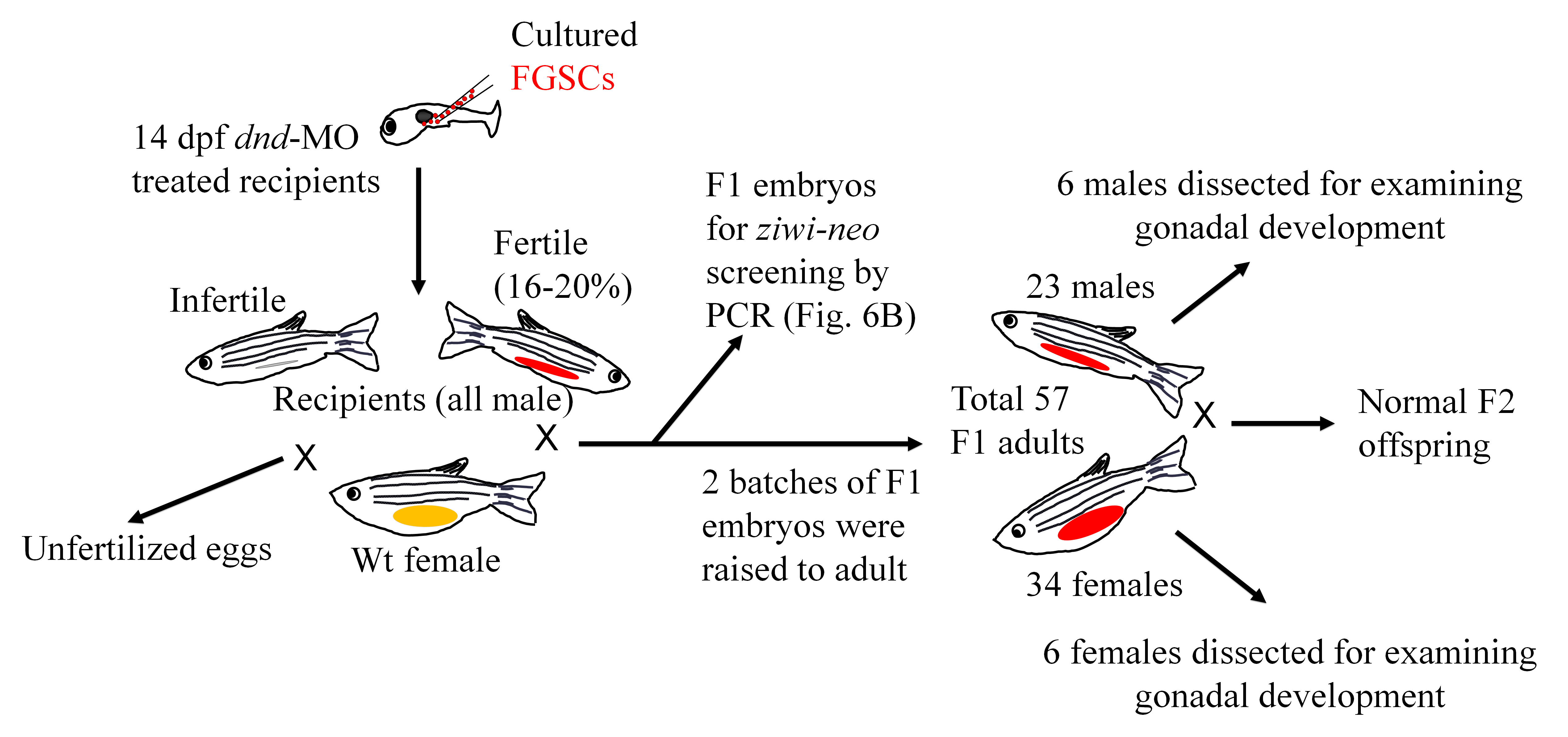

Supplement: Figure S2 — A flow chart diagram of the FGSC transplantations, screenings and analyses. All adult recipients (F0) were found to be males and were able to induce wild-type (Wt) females to spawn eggs; about 16% to 20% of recipients were able to produce fertilized eggs. F1 offspring were screened to confirm the presence of ziwi-neo using PCR (Fig. 6B). An F1 generation produced by transplanting 6-week-old FGSC cultures was raised to adult and confirmed that normal and healthy F1 male and female can be obtained. Normal F2 offspring were produced by crossing F1 males with F1 females. (TIF) [file pone.0062660.s002.tif]
