# Supplementary material for: Production of Zebrafish Offspring from Cultured Female Germline Stem Cells
Source: PLoS One. 2013 May 3;8(5):e62660. doi: 10.1371/journal.pone.0062660 (PMC3643964; doi:10.1371/journal.pone.0062660)
Supplement: Table S1 — Effect of feeder cells expressing zebrafish Lif, Fgf2 and Gdnf on 3-week and 6-week FGSC cultures. Number of FGSCs in each individual colony among 5 different culture conditions was presented. (DOC) [file pone.0062660.s003.doc]

| **Feeders** | **OFC3** | | | | | | |  | **OFC3L** | | | | | | |  | **OFC3LF** | | | | | | |  | **OFC3LG** | | | | | | |  | **OFC3LF+ OFC3LG** | | | | | | |
| --- | --- | --- | --- | --- | --- | --- | --- | --- | --- | --- | --- | --- | --- | --- | --- | --- | --- | --- | --- | --- | --- | --- | --- | --- | --- | --- | --- | --- | --- | --- | --- | --- | --- | --- | --- | --- | --- | --- | --- |
|  |  | | | | | | |  |  | | | | | | |  |  | | | | | | |  |  | | | | | | |  |  | | | | | | |
| **Time of culture** | 3 weeks | | |  | 6 weeks | | |  | 3 weeks | | |  | 6 weeks | | |  | 3 weeks | | |  | 6 weeks | | |  | 3 weeks | | |  | 6 weeks | | |  | 3 weeks | | |  | 6 weeks | | |
| **Wells** | A | B | C |  | A | B | C |  | A | B | C |  | A | B | C |  | A | B | C |  | A | B | C |  | A | B | C |  | A | B | C |  | A | B | C |  | A | B | C |
|  |  |  |  |  |  |  |  |  |  |  |  |  |  |  |  |  |  |  |  |  |  |  |  |  |  |  |  |  |  |  |  |  |  |  |  |  |  |  |  |
|  | 7 | 4 | 6 |  | 4 | 7 | 7 |  | 4 | 7 | 6 |  | 6 | 20 | 4 |  | 13 | 15 | 13 |  | 17 | 9 | 18 |  | 12 | 4 | 9 |  | 18 | 32 | 13 |  | 19 | 6 | 8 |  | 32 | 96 | 49 |
|  | 4 | 4 | 4 |  | 6 | 4 | 4 |  | 7 | 6 | 9 |  | 6 | 11 | 12 |  | 14 | 14 | 14 |  | 36 | 28 | 9 |  | 7 | 7 | 13 |  | 22 | 13 | 11 |  | 4 | 21 | 16 |  | 124 | 33 | 58 |
| ***Cell number** | 6 | 5 | 6 |  | 5 | 12 | 5 |  | 13 | 4 | 11 |  | 14 | 7 | 22 |  | 4 | 7 | 4 |  | 14 | 26 | 24 |  | 14 | 15 | 4 |  | 12 | 24 | 24 |  | 16 | 14 | 4 |  | 25 | 12 | 12 |
| **in each colony** | 4 | 12 | 5 |  | 8 | 11 | 7 |  | 11 | 6 | 8 |  | 26 | 11 | 8 |  | 17 | 10 | 19 |  | 21 | 34 | 27 |  | 17 | 5 | 11 |  | 10 | 9 | 37 |  | 16 | 16 | 16 |  | 47 | 78 | 26 |
|  | 12 | 7 | 9 |  |  | 5 | 10 |  | 10 | 12 | 8 |  | 21 | 20 | 16 |  | 12 | 13 | 10 |  | 27 | 36 | 11 |  | 6 | 8 | 7 |  | 28 | 18 | 5 |  | 12 | 22 | 4 |  | 15 | 132 | 8 |
|  | 4 | 9 | 11 |  |  | 6 | 8 |  | 7 | 4 | 12 |  | 18 | 4 | 26 |  | 9 | 13 | 4 |  | 16 | 19 | 8 |  | 7 | 7 | 13 |  | 11 | 24 | 23 |  | 25 | 13 | 12 |  | 20 | 102 | 42 |
|  | 7 | 10 | 9 |  |  | 9 |  |  | 5 | 16 | 5 |  | 4 | 18 | 18 |  | 5 | 6 | 5 |  | 8 | 10 | 18 |  | 13 | 9 | 7 |  | 29 | 19 | 18 |  | 12 | 12 | 24 |  | 149 | 84 | 36 |
|  | 9 | 9 | 4 |  |  |  |  |  | 9 | 18 | 8 |  | 14 | 16 | 14 |  | 11 | 11 | 10 |  | 41 | 21 | 21 |  | 12 | 14 | 12 |  | 16 | 31 | 21 |  | 7 | 6 | 17 |  | 6 | 46 | 21 |
|  | 5 | 7 | 4 |  |  |  |  |  | 11 | 4 | 12 |  | 10 | 22 | 13 |  | 13 | 12 | 15 |  | 26 | 25 | 35 |  | 9 | 10 | 8 |  | 8 | 11 | 19 |  | 28 | 6 | 7 |  | 22 | 39 | 15 |
|  | 6 | 5 | 4 |  |  |  |  |  | 10 | 6 | 11 |  | 8 | 17 | 6 |  | 8 | 13 | 12 |  | 25 | 36 | 17 |  | 9 | 9 | 9 |  | 4 | 4 | 31 |  | 4 | 22 | 10 |  | 18 | 66 | 45 |
|  | 10 | 4 | 7 |  |  |  |  |  | 4 | 5 | 7 |  | 13 | 8 | 19 |  | 6 | 6 | 4 |  | 16 | 31 | 29 |  | 15 | 11 | 8 |  | 22 | 27 | 19 |  | 13 | 13 | 23 |  | 79 | 52 | 39 |
|  |  | 5 | 11 |  |  |  |  |  | 9 | 11 | 11 |  | 19 | 20 | 21 |  | 10 | 4 | 4 |  | 32 | 19 | 17 |  | 4 | 6 | 4 |  | 6 | 19 | 8 |  | 12 | 21 | 12 |  | 47 | 28 | 22 |
|  |  | 11 | 8 |  |  |  |  |  | 4 | 6 | 4 |  | 22 | 24 | 25 |  | 12 | 14 | 12 |  | 8 | 32 | 32 |  | 7 | 12 | 15 |  | 14 | 25 | 25 |  | 7 | 11 | 9 |  | 36 | 19 | 98 |
|  |  | 4 | 8 |  |  |  |  |  | 4 | 4 | 4 |  | 18 |  | 16 |  | 12 | 12 | 9 |  | 22 | 17 | 16 |  | 12 | 11 | 9 |  | 19 | 34 |  |  | 12 | 8 | 12 |  | 18 | 4 | 56 |
|  |  |  | 4 |  |  |  |  |  | 11 | 4 | 11 |  | 16 |  | 6 |  | 11 | 14 | 11 |  | 18 | 19 | 25 |  | 11 | 9 | 11 |  | 27 | 28 |  |  | 9 | 9 | 12 |  | 4 | 78 | 104 |
|  |  |  | 9 |  |  |  |  |  | 9 | 12 | 9 |  | 22 |  |  |  | 7 | 7 | 9 |  | 31 | 24 | 26 |  | 7 | 6 | 7 |  | 35 | 10 |  |  | 11 | 9 | 6 |  | 18 | 5 | 86 |
|  |  |  |  |  |  |  |  |  | 9 | 9 | 4 |  |  |  |  |  | 14 | 17 | 11 |  | 33 | 28 | 41 |  | 9 | 9 | 4 |  |  | 14 |  |  | 16 | 17 | 13 |  | 76 | 50 | 74 |
|  |  |  |  |  |  |  |  |  | 5 | 4 | 6 |  |  |  |  |  | 9 | 4 | 8 |  |  | 34 |  |  | 6 | 12 | 10 |  |  |  |  |  | 10 | 14 | 7 |  | 10 | 11 | 18 |
|  |  |  |  |  |  |  |  |  | 11 |  | 7 |  |  |  |  |  | 6 | 19 | 7 |  |  | 18 |  |  | 8 | 8 | 4 |  |  |  |  |  | 8 | 9 | 17 |  | 48 | 8 | 27 |
|  |  |  |  |  |  |  |  |  | 7 |  | 11 |  |  |  |  |  | 16 | 7 | 11 |  |  |  |  |  | 17 | 19 |  |  |  |  |  |  | 21 | 16 | 12 |  | 21 | 48 | 65 |
|  |  |  |  |  |  |  |  |  | 9 |  | 4 |  |  |  |  |  | 14 | 19 | 13 |  |  |  |  |  | 8 | 8 |  |  |  |  |  |  | 6 | 4 | 15 |  | 86 |  | 43 |
|  |  |  |  |  |  |  |  |  | 7 |  | 8 |  |  |  |  |  | 9 | 11 | 9 |  |  |  |  |  | 6 | 16 |  |  |  |  |  |  | 7 | 5 | 7 |  | 12 |  |  |
|  |  |  |  |  |  |  |  |  |  |  | 12 |  |  |  |  |  | 17 | 9 | 10 |  |  |  |  |  | 11 | 12 |  |  |  |  |  |  | 16 | 4 | 16 |  | 28 |  |  |
|  |  |  |  |  |  |  |  |  |  |  |  |  |  |  |  |  |  | 8 | 11 |  |  |  |  |  | 7 | 8 |  |  |  |  |  |  | 9 | 11 | 21 |  |  |  |  |
|  |  |  |  |  |  |  |  |  |  |  |  |  |  |  |  |  |  | 4 |  |  |  |  |  |  |  |  |  |  |  |  |  |  | 11 | 5 | 19 |  |  |  |  |
|  |  |  |  |  |  |  |  |  |  |  |  |  |  |  |  |  |  | 7 |  |  |  |  |  |  |  |  |  |  |  |  |  |  | 4 | 19 |  |  |  |  |  |
|  |  |  |  |  |  |  |  |  |  |  |  |  |  |  |  |  |  |  |  |  |  |  |  |  |  |  |  |  |  |  |  |  | 8 | 6 |  |  |  |  |  |
|  |  |  |  |  |  |  |  |  |  |  |  |  |  |  |  |  |  |  |  |  |  |  |  |  |  |  |  |  |  |  |  |  | 13 | 14 |  |  |  |  |  |
|  |  |  |  |  |  |  |  |  |  |  |  |  |  |  |  |  |  |  |  |  |  |  |  |  |  |  |  |  |  |  |  |  | 9 |  |  |  |  |  |  |
|  |  |  |  |  |  |  |  |  |  |  |  |  |  |  |  |  |  |  |  |  |  |  |  |  |  |  |  |  |  |  |  |  |  |  |  |  |  |  |  |
| Total cell number | 74 | 96 | 109 |  | 23 | 54 | 41 |  | 176 | 138 | 188 |  | 237 | 198 | 226 |  | 249 | 276 | 235 |  | 391 | 466 | 374 |  | 234 | 235 | 165 |  | 281 | 342 | 254 |  | 345 | 333 | 319 |  | 941 | 991 | 944 |
| Total colony number | 11 | 14 | 16 |  | 4 | 7 | 6 |  | 22 | 18 | 23 |  | 16 | 13 | 15 |  | 23 | 26 | 24 |  | 17 | 19 | 17 |  | 24 | 24 | 19 |  | 16 | 17 | 13 |  | 29 | 28 | 25 |  | 23 | 20 | 21 |
| Ave. cell number/colony | 6.7 | 6.9 | 6.8 |  | 5.8 | 7.7 | 6.8 |  | 8 | 7.7 | 8.2 |  | 15 | 15 | 15 |  | 11 | 11 | 9.8 |  | 23 | 25 | 22 |  | 9.8 | 9.8 | 8.7 |  | 18 | 20 | 20 |  | 12 | 12 | 13 |  | 41 | 50 | 45 |

(*Colonies contained 4 or more FGSCs were shown and considered as mitotically active FGSC colonies)
